# Correction to “Epiplakin1 promotes the progression of esophageal squamous cell carcinoma by activating the PI3K‐AKT signaling pathway”

**DOI:** 10.1111/1759-7714.15195

**Published:** 2023-12-20

Qiao Z, Dai C, Wang Z, Wang Z, Wang Z, Zhang T, et al. Epiplakin1 promotes the progression of esophageal squamous cell carcinoma by activating the PI3K‐AKT signaling pathway. Thorac Cancer. 2022;13:1117–1125. https://doi.org/10.1111/1759-7714.14366.

There was a mistake in the wound healing assay in Figure 4a. The revised figure is shown below:

**FIGURE 4:**
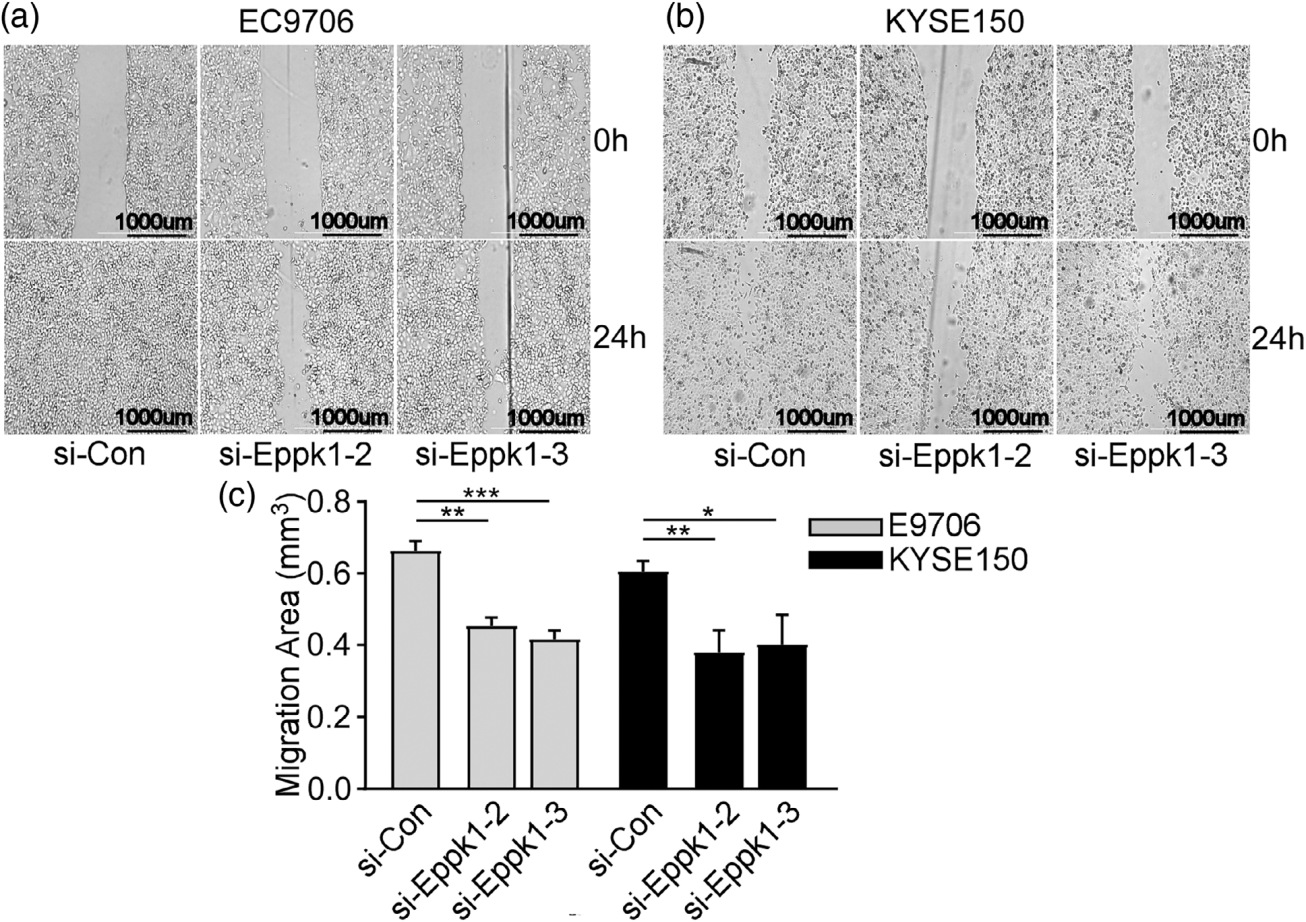
EPPK1 regulated the migration and invasion via EMT progression. (a) The wound healing rate of ESCC cells detected by wound healing assay (*p* < 0.05). (b) The invasion of ESCC cells detected by transwell assay (*p* < 0.05). (c) The proteins related to EMT measured by western blot (*p* < 0.05). Values represent means ± SD, *n* = 3. *p < 0.05, **p < 0.01, ***p < 0.001.

We apologize for these errors.

